# A Novel Intelligent Fault Diagnosis Method for Rolling Bearing Based on Integrated Weight Strategy Features Learning

**DOI:** 10.3390/s20061774

**Published:** 2020-03-23

**Authors:** Jun He, Ming Ouyang, Chen Yong, Danfeng Chen, Jing Guo, Yan Zhou

**Affiliations:** 1 College of Automation, Foshan University, Foshan City 528000, Guangdong Province, China; hejun_723@fosu.edu.cn (J.H.); cheny@fosu.edu.cn (C.Y.); cdf2017@fosu.edu.cn (D.C.); guojing@fosu.edu.cn (J.G.); 2 College of Computer Science, Foshan University, Foshan City 528000, Guangdong Province, China; zhouyan791266@163.com

**Keywords:** stacked auto-encoder, weighting strategy, deep learning, bearing fault diagnosis

## Abstract

Intelligent methods have long been researched in fault diagnosis. Traditionally, feature extraction and fault classification are separated, and this process is not completely intelligent. In addition, most traditional intelligent methods use an individual model, which cannot extract the discriminate features when the machines work in a complex condition. To overcome the shortcomings of traditional intelligent fault diagnosis methods, in this paper, an intelligent bearing fault diagnosis method based on ensemble sparse auto-encoders was proposed. Three different sparse auto-encoders were used as the main architecture. To improve the robustness and stability, a novel weight strategy based on distance metric and standard deviation metric was employed to assign the weights of three sparse auto-encodes. Softmax classifier is used to classify the fault types of integrated features. The effectiveness of the proposed method is validated with extensive experiments, and comparisons with the related methods and researches on the widely-used motor bearing dataset verify the superiority of the proposed method. The results show that the testing accuracy and the standard deviation are 99.71% and 0.05%.

## 1. Introduction

With the upgrading of industrial capacity, the connection between machine equipment is increasingly inseparable. Once unexpected faults happen in a machine, it may indirectly effect the reliability of other connected machineries [1]. These failures will cause heavy economic loss, and even more seriously, they could be life-threatening [2]. Therefore, the automatic, accurate, and timely recognition of the health conditions of machine equipment is highly necessary [3,4]. 

In the past few years, intelligent fault diagnosis methods have attracted great attentions and widely adopted in the condition monitoring systems [5,6]. Generally, intelligent fault diagnosis methods can be divided three main steps: (1) signals acquisition; (2) feature extraction and selection; (3) fault classification [7,8]. After a literature review, it can be found that a tremendous amount of researches have focused on how to extract discriminative features from collected signals based on abundant signal processing technologies [9,10], such as time-domain [11,12], frequency-domain [13], time-frequency-domain statistics analytical methods [14], or other waveform transform methods [15,16]. To classify the extracted features, a few artificial intelligence methods (ANN, SVM, etc.) are applied. For instance, Fu et al. [17] proposed a novel hybrid approach coupling variational mode decomposition and SVM to identify rolling bearing fault types. Ali et al. [15] used empirical mode decomposition to extract 10 time-domain statistical features and an artificial neural network is used to identify the health conditions of rolling bearing. He et al. [18] proposed an ensemble error minimized learning machine method to recognize rolling bearing faults, empirical mode decomposition technology is adopted to extract the ensemble time-domain features. However, although these traditional intelligent methods did work and achieved an accurate diagnosis result, they still have two deficiencies: (1) the features are usually manually extracted depending on prior knowledge and diagnostic expertise, which accorded to a specific fault type and probably unsuitable for other faults [19,20]; (2) In real industries, the collected signals are usually exposed to environmental noises, which cause the signals to be complex and non-stationary, and signal processing technologies need to be employed to filter the collected signals to obtain the effective features [3,21]. Consequently, there is an urgent need to develop new intelligent fault diagnosis methods to accomplish fault diagnosis tasks automatically.

As an emerging research field, deep learning has a powerful ability to extract the representative features from the collected signals, which makes it has the potential to overcome the shortcomings of the traditional intelligent diagnosis methods [22,23]. The advantage of deep learning is that can automatically learn discriminative features and classified faults, which removes the requirements of manual feature extraction and prior knowledge from the diagnosis model. After more than ten years of development, deep learning has been gradually applied to the field of fault diagnosis. For example, Liu et al. [24] presented a fault diagnosis method for rolling bearings based on convolution neural network (CNN) in which the step k is used to discretize the vibration signal and the discrete sequence as the input data of CNN. Jia et al. [25] used the normalized sparse AEs to constitute local connection network, and the model can learn to avoid similar, repeated features and overcome the problem of feature change. Shao et al. [26] proposed an improved convolution deep belief network method based on compressed sensing technology, this method used compressed data as the input of the model and obtained less time consumption of the fault diagnosis. A novel cross-domain fault diagnosis method was proposed by Li et al. [27] whereby multiple deep generative neural networks were employed to generate corresponding-domain fake samples, and faults in different domains could be discriminated well. Long et al. [28] used a competitive swarm optimizer and a local search algorithm to optimize the weights of echo state networks for decreasing the affect caused by random selection of input weights and reservoir weights. Although the above researches are successfully applied in fault diagnosis, there still exist shortcomings in that these intelligent diagnosis methods based on deep learning mainly focus on the research of the individual learning model. Due to complexity of the collected vibration data and even there are exiting the imbalance between different data [29], the generalization can seldom perform well consistently when used individual deep learning model. This problem derives from the limitation of individual deep learning models for the fault diagnosis of complicate mechanical equipment [30]. Ensemble learning is another method of machine learning that can effectively deal with this problem, ensemble learning uses several models and an integration strategy to maximize the strengths of individual models and achieve better results than an individual model [31,32]. Among them, the integration strategy plays an important role in the ensemble learning, and directly affect the accuracy of the diagnosis results. Therefore, it is meaningful to study ensemble learning models. 

In this paper, a novel ensemble learning method based on multiple stacks sparse AEs is proposed for bearing intelligent fault diagnosis. The proposed method is mainly included three steps: Firstly, three stack sparse AEs with different weights are used to extract the representative features from the raw vibration signals. Secondly, a feature integrated strategy based on distance and standard deviation metrics is designed to fine tune the extracted features, which improves the robustness and stability of the diagnosis result. Finally, the softmax classifier is used to classify the fault types based on the integrated features. Experimental results show that the proposed method can get rid of the dependence of manual design algorithm to extract features, and overcome the limitations of an individual deep learning model, which is superior compared with other similar intelligent diagnosis methods. In brief, the contributions of this paper are summarized as follows:

(1) A novel ensemble deep learning method-based multiple stacks sparse AE is proposed for bearing intelligent fault diagnosis. This method is a segmented adaptive feature extraction procedure and can automatically classify the health status of the rolling machinery. Since the proposed method can process three segments of signals at the same time, it is more suitable for processing massive data in the fields of condition monitoring and fault diagnosis.

(2) A feature integrated strategy is designed to assign the weight of each feature. The strategy is composed of distance weight and variance weight, which can decrease the distance of intra-class and increase the distance of inter-class, improving the robustness and stability of fault diagnosis.

(3) A common motor bearing dataset is used to verify the proposed method. In the course of research, the selection of several key parameters and effects of segments and training samples on the diagnosis performance were studied. In addition, this method is compared with different methods and relative similar studies, the results show the superiority of the proposed method.

The remainder parts are organized as follows. In Section 2, the theory of the stack sparse AEs and softmax classifier are briefly introduced. In Section 3, the proposed method is described in detail. Section 4 demonstrates the experiment results on a popular rolling bearing. Conclusions are given in Section 5.

## 2. Stack Sparse Auto-Encoders and Softmax Classifier

### 2.1. Stack Sparse Auto-Encoders

**Figure 1:**
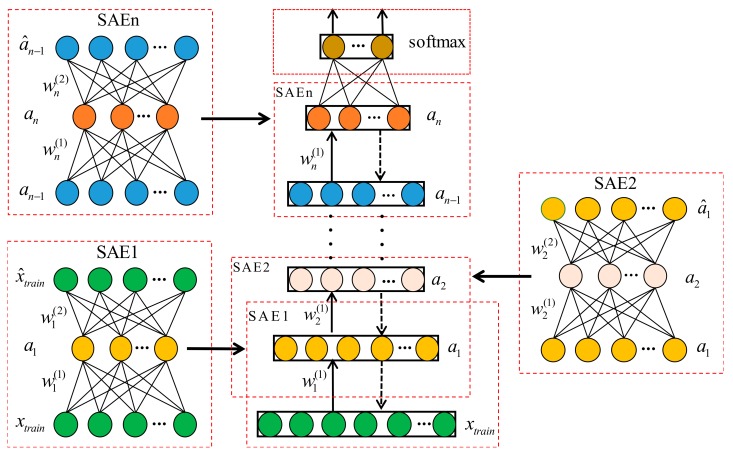
AE structure and the training process of SAE.

In this section, we will briefly introduce the standard stack auto-encoder (SAE). As an unsupervised learning model, SAE has wide application in pattern recognition fields [33]. It consists of several auto-encoders, each of which is a symmetrical three-layer neural network, including encoder network and decoder. The network parameters can be initialized by minimizing the reconstruction error between the input data and the output data [34,35]. Further, the expected SAE can be obtained through layer by layer training, the structure of auto-encoder (AE), and the training process of SAE, as shown in Figure 1.

Suppose a n-dimensional unlabeled training sample is $x=\left\{x_{1}\right.,x_{2},\ldots ,\left.x_{n}\right\}\in {ℜ}^{1\times n}$, the training process of AE is a representation that transform the input sample x into a hidden layer vector $a_{1}$, the vector $a_{1}$ can be denoted as $a_{1}=\left\{a^{1}\right.,a^{2},\ldots ,\left.a^{s}\right\}\in {ℜ}^{1\times s}$, the calculation procedures is as follows:

(1) $$a_{1}=f\left(w_{1}^{\left(1\right)}x+b_{1}^{\left(1\right)}\right)$$

where $w_{1}^{\left(1\right)}$ is the weight matrix, $b_{1}^{\left(1\right)}$ and f(⋅) are the offset vector and the activation function, respectively. Sigmoid [36,37] as the activation function used to train AE given as follows:

(2) $$f(z)=1/\left(1+e^{-z}\right)$$

Then, the hidden vector $a_{1}$ will be decoded and reconstructed as the vector $\hat{x}$ by the Equation (3), the vector $\hat{x}$ can be denoted as $\hat{x}=\left\{{\hat{x}}_{1}\right.,{\hat{x}}_{2},\ldots ,\left.{\hat{x}}_{n}\right\}\in {ℜ}^{1\times n}$. Equation (3) gives as follows:

(3) $$\hat{x}=f(w_{1}^{\left(2\right)}a_{1}+b_{1}^{\left(2\right)})$$

where the $w_{1}^{\left(2\right)}$ and $b_{1}^{\left(2\right)}$ are the parameters of hidden layer to output layer. This works as the Equation (1).

The aims of training process is to obtain the approximation optimal value of parameter w and b through minimized the reconstruction errors. 

For a sample set ${\left\{x^{m}\right\}}_{m=1}^{M}$ with M samples, its reconstruction cost function can be expressed as follow:

(4) $$J_{1}(w,b)=\frac{1}{M}\sum_{m=1}^{m}L(x^{m},{\hat{x}}^{m})$$

where $L(x^{m},{\hat{x}}^{m})$ is the reconstruction error square, which is given as Equation (5)

(5) $$L(x^{m},{\hat{x}}^{m})={\left\|x^{m}-{\hat{x}}^{m}\right\|}^{2}$$

### 2.2. Sparse Auto-Encoder

In the training process of AE, training samples usually contain a lot of redundant information, which means that the training samples only contain a small amount of useful information, and the hidden neurons are not all activated to represent the information of input data, especially when the dimension of input data is less than the number of hidden neurons. Therefore, for each AE, a sparse constraint is adopted to limit the number of activated neurons in the hidden layer [37,38]. Kullback–Leibler (KL) divergence, as a constraint condition usually used in AE training, can be expressed as follows:

(6) $$KL(\rho ||{\hat{\rho }}_{j})=\rho \log\frac{\rho }{{\hat{\rho }}_{j}}+(1-\rho )\log\frac{1-\rho }{1-{\hat{\rho }}_{j}}$$

where ρ and ${\hat{\rho }}_{j}$ are the sparse factor and average activated number of jth hidden neurons, respectively, and $KL(\rho ||{\hat{\rho }}_{j})$ denotes the discrepancy ρ and ${\hat{\rho }}_{j}$.

To sum up the above conclusions, it can minimize the cost function of each AE to get optimal pre-training parameters w and b. So, the cost function can be rewritten as follows:

(7) $$J(W,b)=\frac{1}{M}\sum_{m=1}^{M}L(x^{m},{\hat{x}}^{m})+\beta \cdot \sum_{j=1}^{s}KL(\rho ||{\hat{\rho }}_{j})$$

where β is the dilution penalty factor.

### 2.3. Softmax Classifier

Softmax classifier is a linear classifier that commonly used in multi-classification tasks, whose output is the probability value of each class [39]. Given a training sample set ${\left\{x^{m}\right\}}_{m=1}^{M}$ and $x^{m}\in {ℜ}^{1\times n}$, its corresponding sample label set is ${\left\{y^{m}\right\}}_{m=1}^{M}$ with $y^{m}\in \left\{1,2,\ldots ,K\right\}$. For each given sample $x^{m}$, softmax classifier will compute the values $p(y^{m}=k|x^{m})$, which is the probability of each class. Therefore, for each different input sample, the output is always a K dimension vector of probability, and the position of the maximum probability determines the class of the sample, which can be expressed by the following hypothetical functions

(8) $$h_{\theta }(x^{m})=\left[\begin{array}{l} p(y^{m}=1|x^{m};\theta )\\ p(y^{m}=2|x^{m};\theta )\\ \;\;\;\vdots \\ p(y^{m}=K|x^{m};\theta ) \end{array}\right]=\frac{1}{\sum_{k=1}^{K}e^{\theta_{k}^{T}x^{m}}}\left[\begin{array}{l} e^{\theta_{1}^{T}x^{m}}\\ e^{\theta_{2}^{T}x^{m}}\\ \;\vdots \\ e^{\theta_{K}^{T}x^{m}} \end{array}\right]$$

where $\theta ={\left[\theta_{1},\theta_{2},\ldots ,\theta_{K}\right]}^{T}$ is the parameter of Softmax classifier, $h_{\theta }$ is the normalized probability. The optimization of model parameters can be achieved by minimizing the cost function J(θ).

(9) $$J(\theta )=-\frac{1}{M}\left[\sum_{m=1}^{M}\sum_{k=1}^{K}I\left\{y^{m}=k\right\}\log\frac{e^{\theta_{k}^{T}x^{m}}}{\sum_{k=1}^{K}e^{\theta_{k}^{T}x^{m}}}\right]$$

where $I\left\{\cdot \right\}$ is an indicator function, when the condition is true, the function return 1 otherwise return 0. 

## 3. Proposed Fault Diagnosis Method

**Figure 2:**
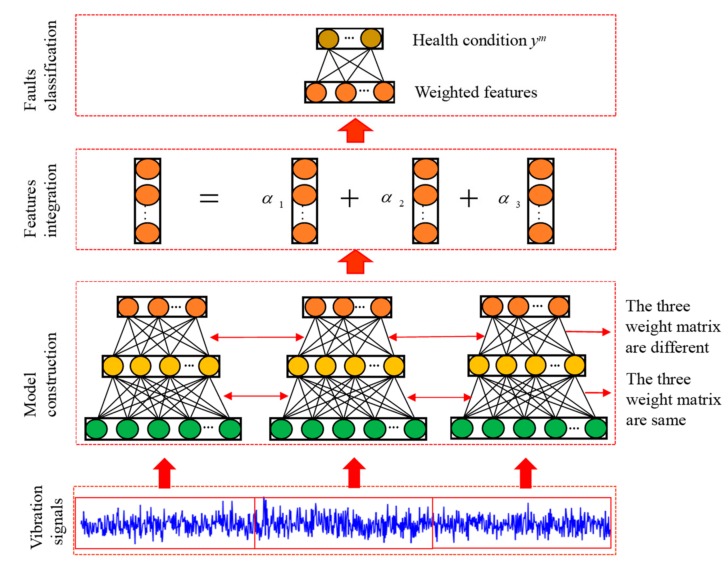
Illustration of the proposed.

In this section, the proposed bearing fault diagnosis method is presented. First, three different sparse auto-encoders are constructed and used to extract the features from the raw vibration signal in Section 3.1. The weight strategy is described in Section 3.2. In Section 3.3, the feature integration is introduced. Softmax classifier is used to classify the health condition of the integrated features, and the detailed process is shown in Figure 2. 

### 3.1. Ensemble Auto-Encoders Construction

**Figure 3:**
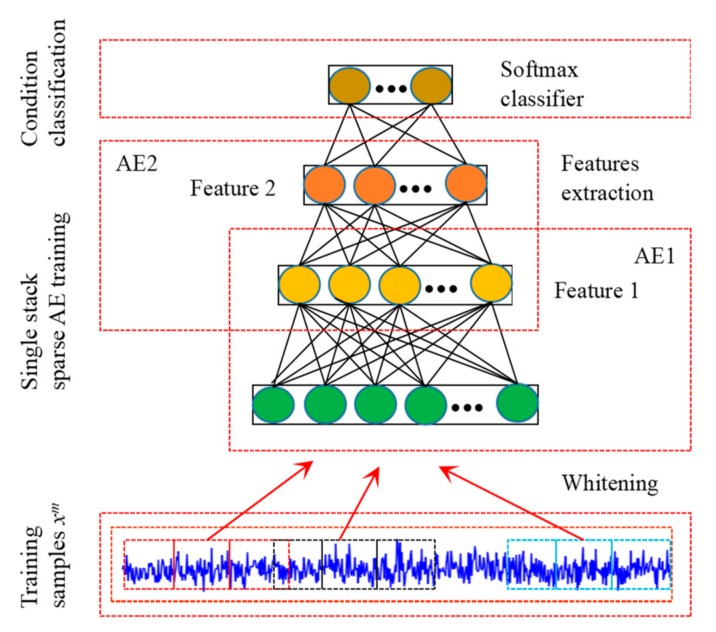
Illustration of the SAE training process.

In order to construct three different sparse auto-encoder models, we divide the original vibration signal into three segments, and each segment uses a SAE to extract features. Assuming the input dimension of SAE is $N_{in}$, when training individual SAE, we randomly select $N_{t}$ training samples from the data set, which are obtained by overlapping sampling method. Each training sample consists of three $N_{in}$ segments, which means that there are three segment samples that can be used to training in each $N_{t}$. The details of training process are shown in the Figure 3.

When the training of individual SAE is completed, removing softmax classifier, and reproduce the parameters of the feature extraction part to other two SAEs. Then, keeping the weight $w_{1}^{\left(1\right)}$ unchanged, and add a small variety to the weight $w_{2}^{\left(1\right)}$ of other two SAEs. This not only can extract representative features from raw data and increase the diversity between features, but it is also beneficial when the input samples are similar. Therefore, the proposed model is very concise, greatly reducing training time and increasing the practicality. In addition, in order to improve the robustness of the model, Gaussian white noise is added to the training samples.

### 3.2. Weighting Strategy

A common ensemble strategy is voting method, which has been wildly applied in different ensemble learning models [40,41]. The voting method includes majority voting and weighting voting [31,32]. The majority voting is used to directly calculate the average value of features, and the advantages of this method are convenience and intelligibility. When machines work in a stable environment or without noise interference, majority voting can get good results for the mechanical fault diagnosis. Different with the majority voting, weighting voting assigns different weight for each feature. Obviously, the majority voting is a special case of the weighting voting. When the working environment of the machine changes or the signal contains a lot of noise, the weighting voting has better performance than the majority voting. There are other ensemble methods for integration features, such as the learning method which outputs features to form a new data set, and learning with a new model [42,43].

**Figure 4:**
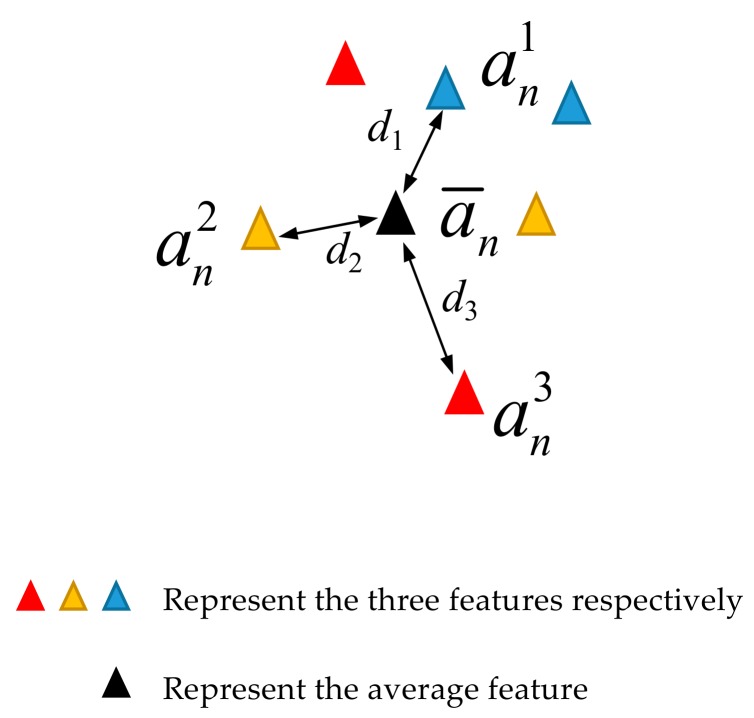
Illustration of the weight selection.

In this paper, in order to improve the robustness and stability of the proposed method, we select the weight voting method to design an integration strategy. As show in Figure 4, assuming that the output features of the three SAEs are $\left\{a_{n}^{1},a_{n}^{2},a_{n}^{3}\right\}$, their distances to the expectation ${\bar{a}}_{n}$ are $d_{1}$, $d_{2}$, $d_{3}$, respectively. When the distance is larger, it means that the feature deviates from the category, and the lower accuracy will be obtained for fault diagnosis. Therefore, we select the distance metric of the three features to the expectation to measure the weight, such as, the larger the distance, the smaller the weight, and vice versa. Suppose the weights of the three SAEs based on distance metric are $\alpha^{\prime }={\left[{\alpha^{\prime }}_{1},{\alpha^{\prime }}_{2},{\alpha^{\prime }}_{3}\right]}^{T}$, mathematically, it can be written as:

(10) $$\alpha^{\prime }=\frac{1}{\sum_{i=1}^{N_{s}}{\left\|a_{n}^{i}-{\bar{a}}_{n}\right\|}_{2}}\left[\begin{array}{l} \;\;\sum_{i=2}^{N_{s}}{\left\|a_{n}^{i}-{\bar{a}}_{n}\right\|}_{2}\\ \sum_{i=1,i\neq 2}^{N_{s}}{\left\|a_{n}^{i}-{\bar{a}}_{n}\right\|}_{2}\\ \sum_{i=1,i\neq 3}^{N_{s}}{\left\|a_{n}^{i}-{\bar{a}}_{n}\right\|}_{2} \end{array}\right]$$

where $N_{s}$ denotes the number of input segments; n is the n*th* hidden layer; ${\left\|\cdot \right\|}_{2}$ is the Euclidean distance; denominator $\sum_{i=1}^{N_{s}}{\left\|a_{n}^{i}-{\bar{a}}_{n}\right\|}_{2}$ is to normalize the weight distribution.

It should be noticed that, although the distance metric can constrain the feature deviation from the average feature on the same faulty category, when the feature itself has a large deviation, the distance metric may not have a good function. Based on this, we introduce the second weight measure condition: standard deviation metric. Standard deviation can reflect the degree of data fluctuation, the larger the standard deviation gets, the greater the data fluctuation is. So, the standard deviation can reflect the stability of features. Suppose that the weights of the three SAEs based on standard deviation metric are $\alpha^{\prime \prime }={\left[{\alpha^{\prime \prime }}_{1},{\alpha^{\prime \prime }}_{2},{\alpha^{\prime \prime }}_{3}\right]}^{T}$, they are defined by:

(11) $$\alpha^{\prime \prime }=\frac{1}{\sum_{i=1}^{N_{s}}\rho_{i}}\left[\begin{array}{l} \;\sum_{i=2}^{N_{s}}\rho_{i}\\ \sum_{i=1,i\neq 2}^{N_{s}}\rho_{i}\\ \sum_{i=1,i\neq 3}^{N_{s}}\rho_{i} \end{array}\right]$$

where ρ denotes the standard deviation of each feature; Denominator $\sum_{i=1}^{N_{s}}\rho_{i}$ is to normalize the weight distribution.

Now we have two feature-related weight vectors, distance metric weight and standard deviation metric weight. To implement an excellent integration strategy, we assume the target weight $\alpha ={\left[\alpha_{1},\alpha_{2},\alpha_{3}\right]}^{T}$, it is defined as follow:

(12) $$\alpha =\lambda \alpha^{\prime }+\gamma \alpha^{\prime \prime }$$

where λ and γ are two hyper-parameters by user-specifying, which the limits of the values are between 0 and 1 and their sum is 1. In the proposed method, the two hyper-parameters will be studied in detail for the effect of diagnosis performance. 

### 3.3. Feature Integration

After the above analysis, the weight vector $\alpha ={\left[\alpha_{1},\alpha_{2},\alpha_{3}\right]}^{T}$ can be determined for each sample $x^{m}$. Meanwhile, three feature vectors $\left[f_{1},f_{2},f_{3}\right]$ are extracted from the input sample $x^{m}$ by the three SAEs. The final object features $f^{m}$ are aggregated using the weight strategy, which is written as follows:

(13) $$f^{m}=\alpha_{1}f_{1}+\alpha_{2}f_{2}+\alpha_{3}f_{3}$$

**Figure 5:**
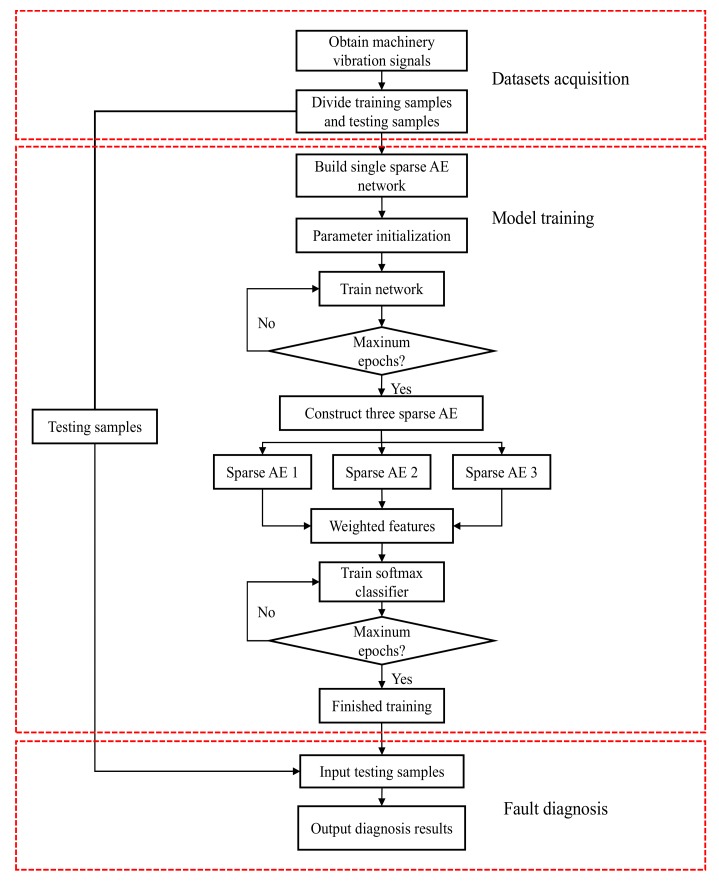
Flow chat of the proposed bearing fault diagnosis method.

This weighted strategy is beneficial that can decrease the influence of the random features caused by ambient noise and interference. Also, the weighted way enhances the discriminative features that these features are complementary and improves the stability due to having the weight constraint term. The detailed process of the proposed method given as Figure 5. 

## 4. Experiment and Analysis

### 4.1. Dataset Description

**Figure 6:**
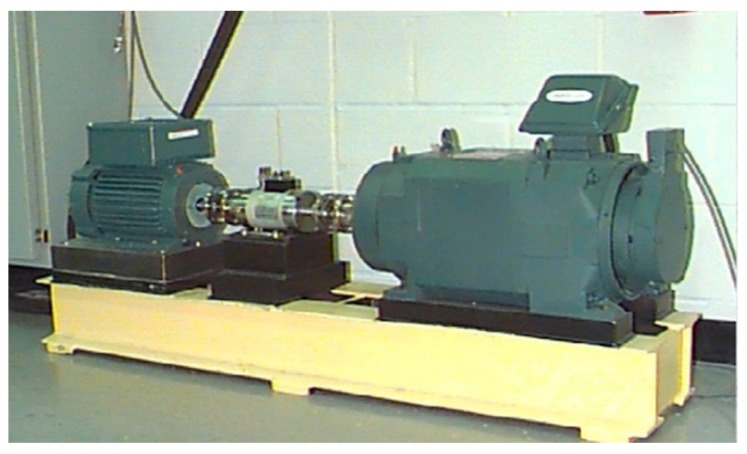
Bearing platform used for experiment.

The bearing dataset provided by Case Western Reserve University [44] is analyzed in this section. As show in Figure 6, the test rig main consists of a 2-horsepower (hp) motor, a torque converter/encoder, a dynamometer and a control circuit. The vibration data were collected from the drive end of a motor under four different conditions: normal condition, inner race fault (IF), roller fault (RF), and outer race fault (OF). Single point faults were introduced of the motor with fault diameters of 0.18 mm, 0.36 mm, and 0.54 mm, respectively. The bearing data were all collected under four load conditions (0, 1, 2, and 3 hp) with the sampling frequency of 12 kHz.

These vibration data compose the motor bearing dataset, which is used to verify the effectiveness of the proposed method. These data contain ten bearing health conditions under four loads, where the same health condition under different loads is defined as one class. The details of the experimental condition are summarized in Table 1. In this experiment, the first 120,000 points of the vibration data are selected as the preprocessed data under each condition. These preprocessed data are divided into training set and test set.

### 4.2. Compare Studies

**Figure 7:**
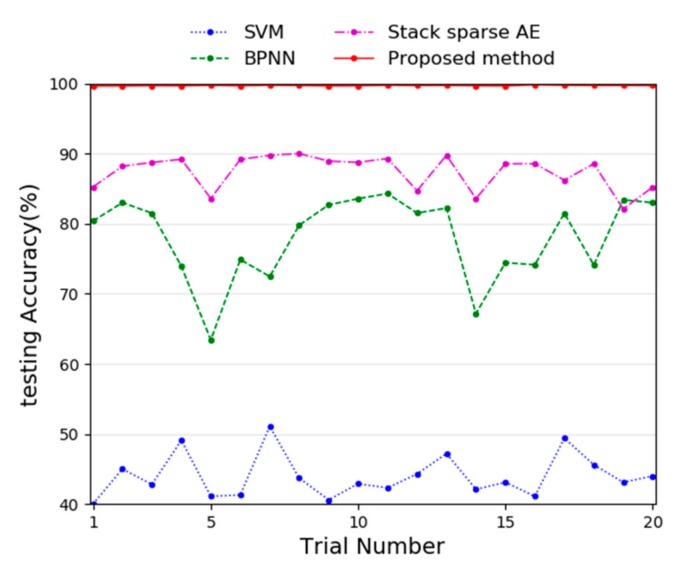
Comparison of different method with 20 experiments.

In order to verify the superiority of the proposed method, three methods were selected to compare with the proposed method, namely, Support Vector Machine (SVM), Back-Propagation Neural Network (BPNN) with two hidden layers, and the individual stack sparse AE with two hidden layers. They are widely used in fault diagnosis of rotating machinery. The input data is raw vibration data, and the comparison of diagnosis performance of the three methods under 20 experiments is shown in Figure 7.

In the Figure 7, the results show that the proposed method has the highest diagnostic accuracy and the smallest fluctuations. Compared with the proposed method, the individual stack sparse AE has smaller diagnosis accuracy and greater diagnosis fluctuation, which indicates that the proposed ensemble method has better diagnosis performance than an individual stack sparse AE. Of course, the individual stack sparse AE is better than the other two diagnosis methods. Since the BPNN is not pre-trained like the individual stack sparse AE, it is under fitting, this proves that AE can reduce the number of training samples. SVM has minimal diagnosis accuracy, because SVM is not suitable for processing high-dimensional data, usually, it needs to preprocess the original vibration data and transform them into statistical features. The specific diagnosis results are summarized in Table 2.

From the perspective of the average accuracy in Table 2, the proposed method shows the best marks with the highest average accuracy (99.71%), while the SVM has the worst diagnosis performance (43.99%), and the individual stack sparse AE only gets intermediate testing accuracy (87.40%). In addition, from the perspective of stability, the proposed method has the optimum performance, with the smallest standard deviation (0.05%), and the BPNN with two hidden layer has the worst performance, with the largest standard deviation (5.91%).

For further proving the superiority of the proposed method, we also compared with other similar studies used the same dataset. In [45], a method adopting 15 stack sparse AEs to extract bearing features was proposed. The 15 stack sparse AEs use different activation function, and the extracted features are integrated with an accuracy threshold. This proposed method classifies the health conditions of 12 motor bearings at 0 hp, and finally obtained an average test accuracy of 97.18% and a standard deviation of 0.11%. Sun et al. [46] proposed a method based on compressed sensing theory. Their method combined with stack sparse AEs to extract features from the compressed data which were used to represent seven bearing health conditions under the load 2. The fault recognition rate of this method is 97.47% and the standard deviation is 0.43% in the bearing database. Lei et al. [47] proposed a bearing diagnosis method to integrate 12 sparse filter networks. The method used a simple average weighted combination strategy to process 12 local features that extracted from raw vibration data and white Gaussian noise is added during training. The method achieved 99.66% diagnosis accuracy and 0.19% standard deviation under 10 fault types and 4 different loads. In method [48], a dynamic weighted average method is designed to aggregate these learned features. This method used three different deep auto-encoder to extract the features, and the accuracy of k-fold cross-validation is used as a metric to assign the weights of the three deep auto-encoders. They obtained the accuracy of 99.69% and standard deviation of 0.24%. Comparing with the above methods, the proposed method in this paper achieved the highest accuracy of fault identification and the smallest standard deviation. The results of the above comparison are displayed in Table 3.

### 4.3. Visualization of Learned Representation

In this section, to qualitatively illustrate the effectiveness of the proposed fault diagnosis method, we visualize the features using four methods. The other three methods are sparse AEs with two hidden layers, the proposed model without whitening method, and the weight average method, respectively. The visual features are extracted from testing sample by the four methods, and the experiment conducted under the condition of noise for a better visual comparison of the result.

A technique called ‘t-SNE’ is used to map the extracted features into a two-dimensional space to achieve visualization of high-dimensional data [49]. This technique has two processes, firstly, the principal component analysis (PCA) is used to reduce the dimension of the features to 50. Then, a technology called ’t-SNE’ is used to represent the 50-dimensional data as two-dimensional planar data.

**Figure 8:**
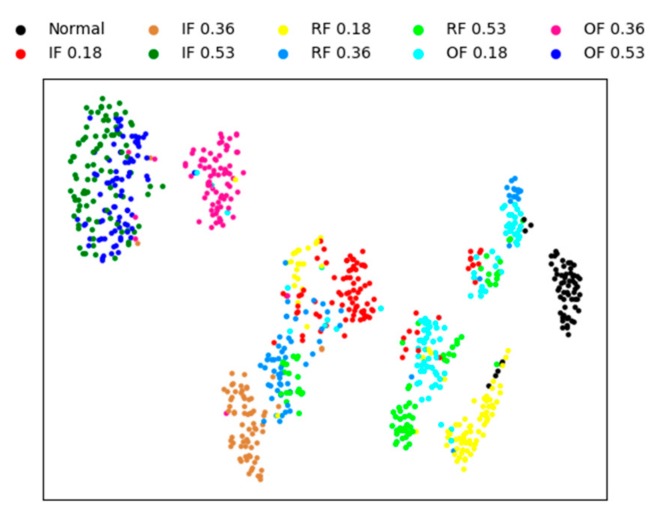
Visualization of sparse AE for the learned features.

Figure 8 is the feature visualization of individual sparse AEs with two hidden layers. It can be seen from the figure that the individual sparse AE method performs aggregation poorly on different fault types. this method cannot correctly diagnose the bearing fault, and only 82% of the test accuracy is obtained.

**Figure 9:**
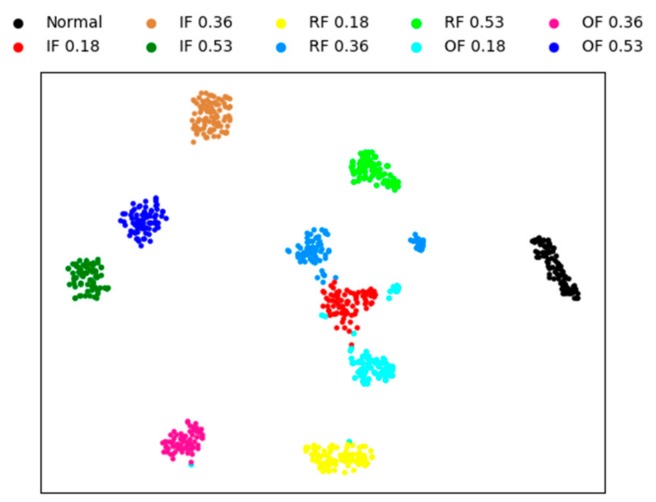
Visualization of without whitening for the learned features.

Figure 9 is the feature visualization of the proposed method without whitening. Comparing with Figure 8, it can be noticed that most of the testing data are clustered in their own category and different types of faults are scattered in different regions. In Figure 9, there are only less intersection between the different fault classes, and the mainly error of fault diagnosis is concentrated in IF 0.18, that is mean that the proposed method cannot completely classify IF 0.18. The distances between different classes are far away, which also shows that the proposed method is robust. The final test accuracy of this method is 96.93%.

**Figure 10:**
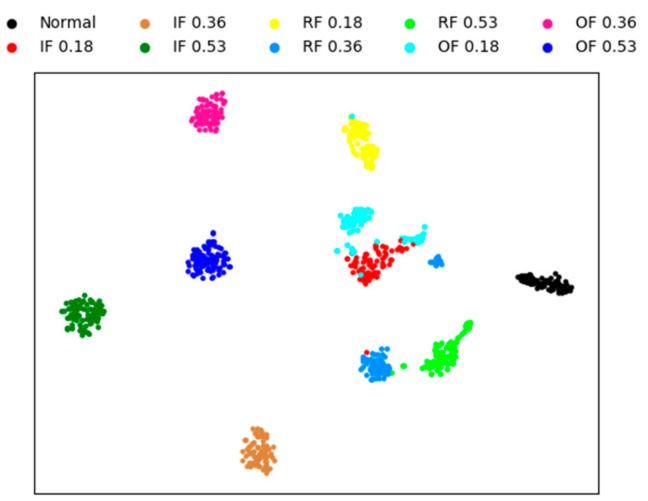
Visualization of weight average for the learned features.

**Figure 11:**
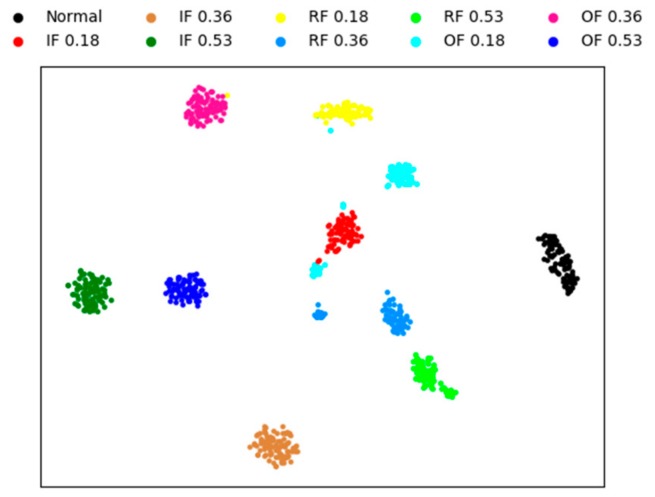
Visualization of proposed method for the learned features.

Figure 10 and Figure 11 shows the feature visualization of the weight average method and our method, respectively. Figure 10 and Figure 11 are very similar, which verifies that the features extracted by the three SAEs are similar, weighting strategy only a fine-tuning operation . Although the difference is not great, the proposed method has better performance than the weight average method, with the fault identification accuracy of 97.78% and 98.23% respectively obtained.

### 4.4. Parameters Selection of the Proposed Method

There are several key parameters need to determine in the proposed method, such as: the input dimension of SAE, the number of hidden layer neurons and sparse parameters ρ, etc. Next, we will respectively investigate the selection of these parameters. In addition, in order to reduce the influence of the randomness, 20 trials are repeated for each experiment. The environment of all experiments are 4G RAM and python 3.6.

**Figure 12:**
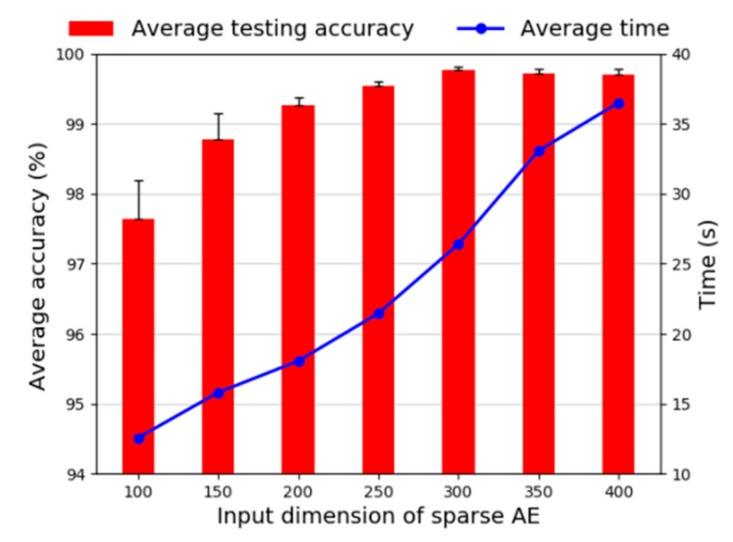
Diagnosis accuracy of various input dimension.

First, we investigate the selection of the input dimension. We select a certain number of samples to train the proposed method, where 40,000 samples are sampled from the bearing dataset, and the rest samples are used for testing. For each trial of different input dimension, we always keep other parameters unchanged. The diagnosis results are displayed in Figure 12, wherein the positive error bars show the standard deviations and the point of time are the average time. It can be seen that when the input dimensions are increasing from 100 to 300, the accuracies are going higher, and when the input dimension is 300, the standard deviation is the smallest. When the input dimension is greater than 300, the average test accuracy only decreases slightly, but the time consumption is growing linearly. Therefore, considering the results from the experiment, we choose 300 as the input dimension.

**Figure 13:**
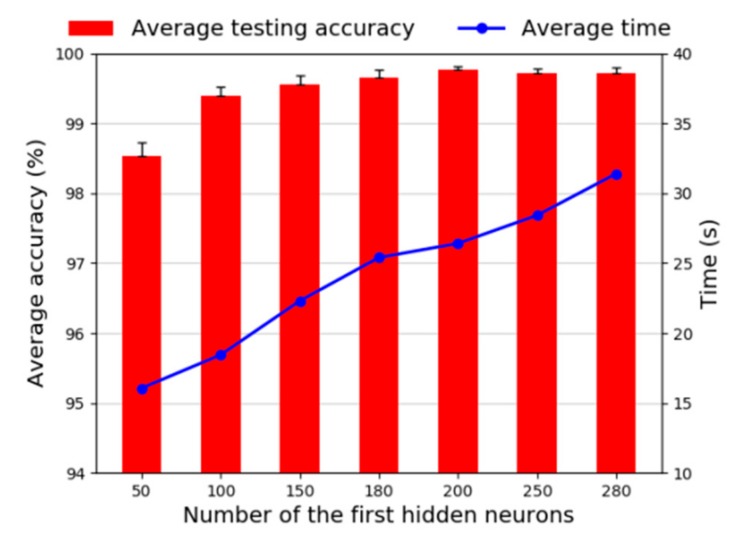
Diagnosis accuracy of neurons in the first layer.

Next we investigate the number of the first hidden layer neurons. As shown Figure 13, the fault recognition accuracy increases gradually, and standard deviation is also reduced as the number of neurons in the first hidden layer increases from 50 to 200. When the number of neurons is greater than 200, the accuracy is stable and corresponding standard deviations are higher. The average time is also increasing. So, we choose 200 as the number of the first hidden layer neurons.

**Figure 14:**
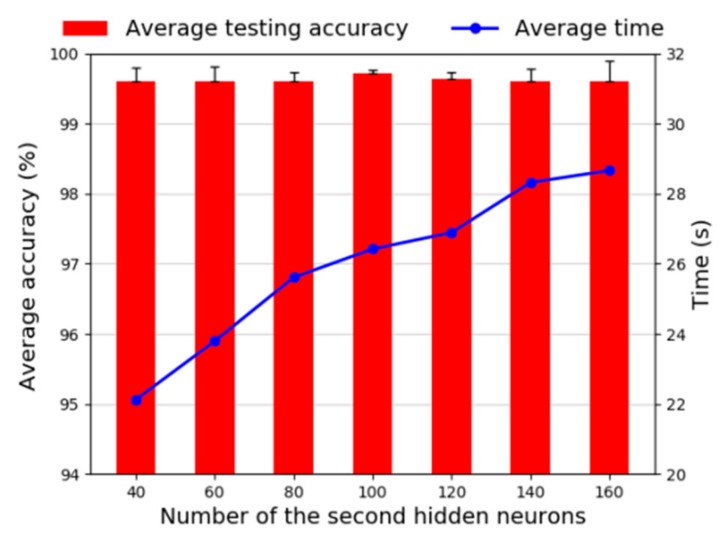
Diagnosis accuracy of neurons number in the second layer.

Then, we investigate the number of the second hidden layer neurons. Generally, the number of neurons in the second hidden layer is less than the first hidden layer. Therefore, the number of neurons we studied was between 40 and 200, and the diagnosis results are shown in Figure 14. It can be seen that the accuracy varies only slightly in the whole neural unit interval. When the number of neurons is 100, the average testing accuracy is highest and standard deviation is smallest. Although time consumption has increased, the increasing values are far from acceptable. Therefore, we choose 100 as the number of the second hidden layer neurons.

**Figure 15:**
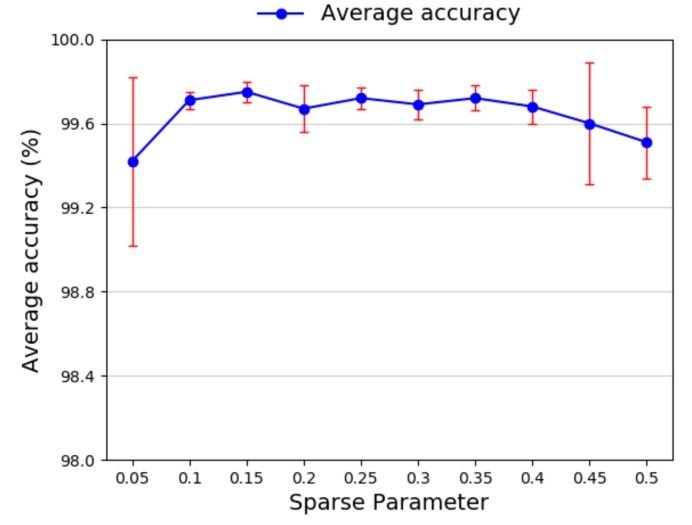
Diagnosis accuracy of different sparse parameters.

Afterward we investigate the selection of sparse parameter ρ. The sparse parameter plays an important role in the process of achieving high accuracy. In general, it is a small value close to zero. According to the general experiment results, the selection of sparse parameter varying from 0.05 to 0.5 is studied. Figure 15 shows the average diagnosis accuracy whit different sparse parameters. It can be seen from the figure that, as the value of sparse factor is 0.15, the highest average test accuracy and smallest standard deviation are obtained. Therefore, 0.15 is chosen as the value of the sparse parameter.

**Figure 16:**
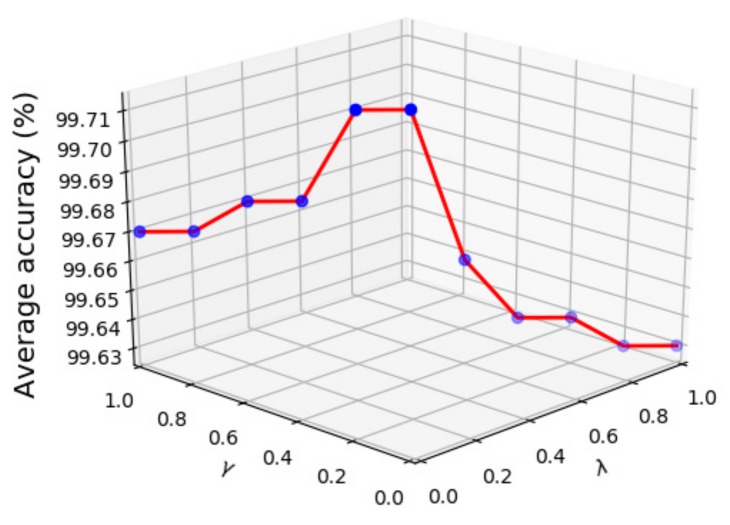
The relationship between average accuracy and parameters λ, γ.

Finally, we investigate the selection of parameters λ and γ. These two parameters are the hyper-parameters of distance metric and standard metric. The diagnosis results are shown in Figure 16, where the two hyper-parameters λ and γ are selected as [0,0.1,0.2,0.3,0.4,0.5,0.6,0.7,0.8,0.9,1] and [1,0.9,0.8,0.7,0.6,0.5,0.4,0.3,0.2,0.1,0], respectively. It can be known that when the model is determined, the average diagnosis accuracy slightly changes as the two parameters correspond to different values, which means that the two hyper-parameters only have the function of fine-tuning. The highest accuracy can be obtained when the parameter λ is between 0.4 and 0.5. Therefore, it is reasonable that λ chooses 0.4 or 0.5. Furthermore, when the parameter γ is greater than λ, the average accuracy is higher, which indicates that the proposed method prefers to choose the standard deviation metric and the distance metric has a smaller impact. 

In summary, the detailed parameters of the proposed method are as follows, the input dimension of sparse AE is 300, the number of two hidden layer neurons is 200, 100, respectively, and the value of sparsely parameter ρ is 0.15, the two hyper-parameters λ γ are 0.5. A wider selection of these parameters in the proposed method are listed in Table 4.

### 4.5. Effect of Segments and Training Samples

The proposed model involves different number of segments and training samples, i.e., a different number of segments for training input and the percentage of training samples for training the proposed model will both significantly impact the diagnosis accuracy and time consumption of the proposed method. Therefore, we study the effect of different number of segments and training samples. 

**Figure 17:**
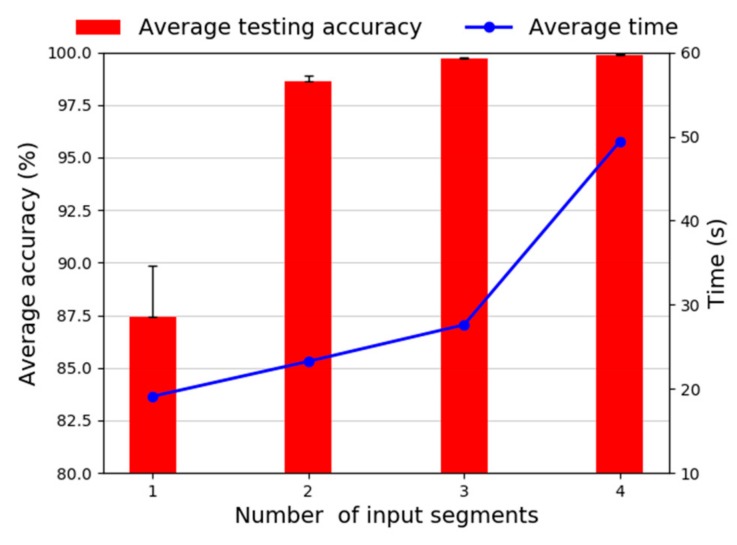
Diagnosis performance with different segments from 1 to 4.

**Figure 18:**
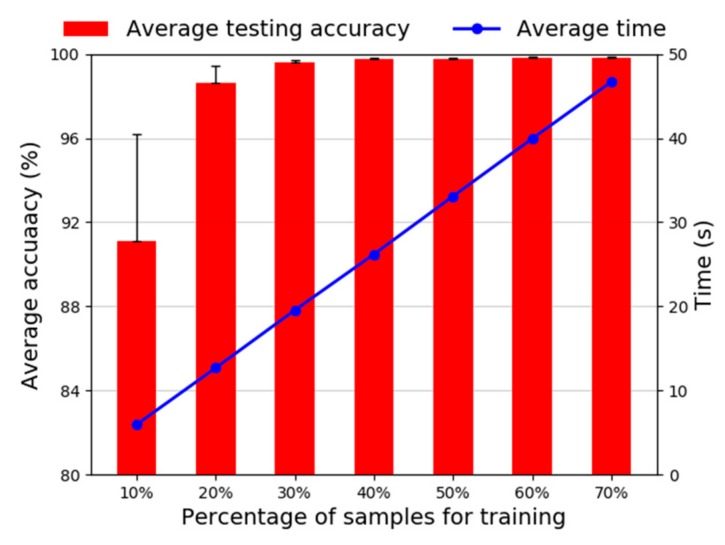
Diagnosis accuracy of different percentage of training samples.

(1) Effect of segments: Different segments determine the structure and diagnosis performance of the model. In this study, signals with different segments will be used as the input for the proposed framework. In order to quantitatively evaluate the effect of the input segments on the classification performance, different segments ranging from one to four are studies. Figure 17 shows the diagnosis accuracy and training time choosing various segments. It is easily observed that when the segment number goes from one to four, the superior diagnosis performance is obtained. The results indicate that the more segments are used, the proposed model can achieve better and more stable performance, because these extracted features from different segments are rich and complementary, it is helpful for classification. Furthermore, a significant accuracy increase and standard deviation decrease from one segment to two segments can be noticed. More segments can achieve better diagnosis performance, however, in reality, it does not mean that more segments are always beneficial, from the comprehensive consideration of the model complexity and computational cost, choosing three segments are reasonable. Table 5 lists the diagnosis performance of different segments corresponding to Figure 17. This result validates that the proposed method can extract more discriminative and stable features from raw vibration signals.

(2) Effect of training samples: In general, as more samples are used to train the model, the higher accuracy can be achieved. The diagnosis results using different percentage of training samples are shown in Figure 18. It can be seen that when the training samples goes larger, the average test accuracy is higher, and the standard deviation is smaller. However, the time consumption is increasing linearly. It means that the selection of training samples is a trade-off between the diagnosis accuracy and the time consumption. The same is true for Figure 12, Figure 13 and Figure 14 and Figure 17. In Figure 18, when the proportion of training samples is 40%, the average test accuracy is 99.71% and the standard deviation is only 0.05%, which means that our proposed method achieves very high diagnosis accuracy and has good stability. 

### 4.6. Robustness Against Environmental Noises

**Figure 19:**
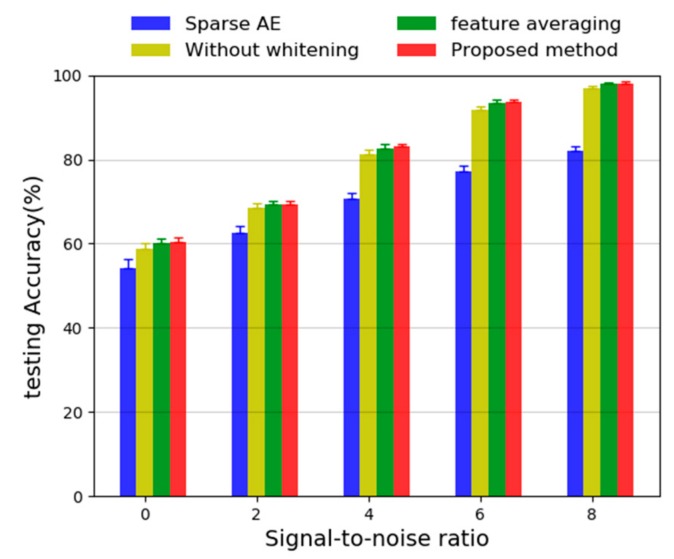
Diagnosis results with environmental noises by different methods.

In the actual industrial production process, noise is everywhere. The raw vibration signals are collected often contain a lot of noise, which has complex variability. For all possible noise, we can’t get all the label samples corresponding to noises. So, in this section, we will study the effect of noise on diagnosis performance by adding Gaussian white noise. The robustness of the proposed method against environmental noise is verified by adding noise to the test data based on the original experiments. Specifically, the noise data is generated by adding Gaussian white noise with different signal-to-noise ratio (SNR) to the test data. The signal-to-noise ratio is defined as

(14) $$SNR=10\log_{10}(\frac{P_{signal}}{P_{noise}})$$

where *P_signal_* and *P_nosie_* represent the power of the original signal and added noise, respectively, the unit of SNR is dB. In this study, we evaluate the proposed method adding noisy signals with different SNR ranging from 0 dB to 8 dB. The results are shown in Figure 19.

It can be seen from the figure that when the SNR increases from 0 dB to 8 dB, the test accuracy of the four methods is increasing. Among them, at each SNR, the proposed method has the highest accuracy and the smallest deviation, the second is the method that using feature averaging method and next is the method without adding Gaussian white noise to the training data, the individual sparse AE with two hidden layer gets worst performance. Compared with the individual sparse AE, it is obvious that the proposed method has better anti-noise performance. In addition, it can be noticed that the proposed method is only slightly better (it is about 0.5%) than the feature average method; it infers that the input data has the same distribution, also, the feature average method can be considered as a sample mean filter, thus random noises will be filtered to some extent. Maybe, when the vibration signals are collected by multiple sensors or the distribution of input data is different, the proposed method may achieve better performance.

## 5. Concluding Remarks

In this paper, a novel bearing fault diagnosis method based on ensemble stack sparse auto-encoder was proposed. A common bearing data set is used, and a large number of experiments are carried out to verify the effectiveness of the proposed method. This paper studies the selection of several key parameters and the influence of segments and training samples on the diagnosis performance. By a comparison with other methods and related studies using the same data set, the superiority of the proposed method is proved. Additionally, the robustness of the proposed method against environmental noises is demonstrated under different levels of noise.

Future research will be extended to other complex models and other fault diagnosis problems such as using a CNN model and remaining useful-life prediction for rolling bearings. In addition, although the proposed method in this paper obtained a high accuracy of fault recognition, it did not achieve satisfactory results in a noisy environment, and there is still much room for improvement. This is one of the future research directions on how to improve the anti-noise performance of the model.

## Figures and Tables

**Table 1:** Bearing data information used to experiment in this proposed.

Fault Type	Fault Size (mm)	Load(hp)	Label
Normal	0.0	0,1,2,3	1
IF	0.18	0,1,2,3	2
IF	0.36	0,1,2,3	3
IF	0.53	0,1,2,3	4
RF	0.18	0,1,2,3	5
RF	0.36	0,1,2,3	6
RF	0.53	0,1,2,3	7
OF	0.18	0,1,2,3	8
OF	0.36	0,1,2,3	9
OF	0.53	0,1,2,3	10

**Table 2:** Experimental results of average accuracy and standard deviation of various methods.

Method	Average Accuracy	Standard Deviation
SVM	43.99%	3.09%
BPNN	78.07%	5.91%
SAE	87.40%	2.44%
Our method	99.71%	0.05%

**Table 3:** Performance comparison with various studies.

Method	Load(hp)	No. of Health Condition	Testing Accuracy	Standard Deviation
[45]	0	12	97.18%	0.11%
[46]	2	7	97.41%	0.43%
[47]	0,1,2,3	10	99.66%	0.19%
[48]	0,1,2,3	10	99.69%	0.24%
Proposed	0,1,2,3	10	99.71%	0.05%

**Table 4:** Key parameters of the proposed method.

Parameters Description	Value
The dimension of Sparse AE	300
The number of the hidden layers	2
The number of the first hidden neurons	200
The number of the second hidden neurons	100
Learning rate	0.007
Sparse parameter	0.15
Sparse penalty factor	2
Batch size	100
Hyper-parameters (λ, γ)	0.5

**Table 5:** Diagnosis accuracy and time consuming for different segments.

Segments	Average Accuracy	Standard Deviation	Training Time (s)	Testing Time (s)
1	87.40%	2.44%	19.11	0.28
2	98.62%	0.23%	23.27	0.36
3	99.71%	0.05%	27.62	0.44
4	99.88%	0.04%	49.44	0.50
